# The effect of top‐predator presence and phenotype on aquatic microbial communities

**DOI:** 10.1002/ece3.2784

**Published:** 2017-02-08

**Authors:** Karen E. Sullam, Blake Matthews, Thierry Aebischer, Ole Seehausen, Helmut Bürgmann

**Affiliations:** ^1^ Department of Surface Waters—Research and ManagementCenter for Ecology, Evolution and BiogeochemistryEawagSwiss Federal Institute of Aquatic Science and TechnologyKastanienbaumSwitzerland; ^2^ Zoological InstituteUniversity of BaselBaselSwitzerland; ^3^ EawagAquatic Ecology DepartmentCenter for Ecology, Evolution and BiogeochemistryKastanienbaumSwitzerland; ^4^ EawagDepartment of Fish Ecology and EvolutionCenter for Ecology, Evolution and BiogeochemistryKastanienbaumSwitzerland; ^5^ Aquatic Ecology and EvolutionInstitute of Ecology & EvolutionUniversity of BernBernSwitzerland; ^6^ Department of BiologyUniversity of FribourgFribourgSwitzerland

**Keywords:** bacterial community composition, ecosystem effects, *Gasterosteus aculeatus*, mesocosms, stickleback

## Abstract

The presence of predators can impact a variety of organisms within the ecosystem, including microorganisms. Because the effects of fish predators and their phenotypic differences on microbial communities have not received much attention, we tested how the presence/absence, genotype, and plasticity of the predatory three‐spine stickleback (*Gasterosteus aculeatus*) influence aquatic microbes in outdoor mesocosms. We reared lake and stream stickleback genotypes on contrasting food resources to adulthood, and then added them to aquatic mesocosm ecosystems to assess their impact on the planktonic bacterial community. We also investigated whether the effects of fish persisted following the removal of adults, and the subsequent addition of a homogenous juvenile fish population. The presence of adult stickleback increased the number of bacterial OTUs and altered the size structure of the microbial community, whereas their phenotype affected bacterial community composition. Some of these effects were detectable after adult fish were removed from the mesocosms, and after juvenile fish were placed in the tanks, most of these effects disappeared. Our results suggest that fish can have strong short‐term effects on microbial communities that are partially mediated by phenotypic variation of fish.

## 1 Introduction

Top predators can affect the biodiversity, composition, and structure of lower trophic levels (Letnic, Ritchie, & Dickman, 2012; Sergio, Newton, & Marchesi, 2005) over a broad range of ecosystem types (Croll, Maron, Estes, Danner, & Byrd, 2005; Paine, 1980; Schmitz, Krivan, & Ovadia, 2004). They can alter prey population structure and resource availability through trophic cascades (Frank, Petrie, Choi, & Leggett, 2005) or modify rates of prey movement, grazing, activity, dispersal, and colonization (Reynolds & Bruno, 2013; Schmitz et al., 2004). Such top predator‐mediated effects can also influence primary producers and bacteria, and may, ultimately, alter biogeochemical cycles, ecosystem processes and abiotic conditions (Chapin et al., 2000; Hooper et al., 2005; Katz et al., 2009; McIntyre, Jones, Flecker, & Vanni, 2007).

In addition to the presence of top predators, the species identity of predators can have differential effects on the species distribution of lower trophic levels (Nilsson et al., 2008) depending on their mode of predation (Biggs et al., 2000; Borer et al., 2005). Recent work suggests that even closely related and phenotypically similar predators can differentially shape community dynamics and ecosystem processes (Bassar et al., 2010; Harmon et al., 2009; Matthews, Hausch, Winter, Suttle, & Shurin, 2011; Post & Palkovacs, 2009). Slight variations in behavioral and life‐history traits that exist between different populations of the same species could potentially influence food‐web structure and ecosystem processes (Moya‐Laraño, 2011; Schmitz, 2009; Wolf & Weissing, 2012).

Morphological or behavioral plasticity can also modify the combined effects of a predator's genetic and environmental background or its phenotype (Pigliucci, 2001; West‐Eberhard, 1989). Differences in rearing environments can cause changes in an animal's morphology, physiology, or behavior that persist over their lifetime, regardless of environmental conditions experienced in adulthood (Kihslinger & Nevitt, 2006; Monaghan, 2008; Smith‐Gill, 1983). For example, animals exposed to different environments may develop differences in learned behavior, resulting in behavioral plasticity and phenotypic variation in predator effects (Ghalambor et al., 2015). Few studies have investigated how both genetic and plastic differences among closely related fish populations affect the composition of communities (Lundsgaard‐Hansen, Matthews, & Seehausen, 2014; Matthews, Aebischer, Sullam, Lundsgaard‐Hansen, & Seehausen, 2016). In general, the focus of these previous studies has been on prey communities, but studying the response of microbial communities is warranted because many of the ecosystem effects of fish might be driven by changes in microbially mediated biogeochemical processes.

Previous work studying fish effects on microbial communities have largely focused on their impact on bacterial production, the synthesis of new biomass by heterotrophic bacterioplankton, or respiration (Christoffersen, Riemann, Klysner, & Sondergaard, 1993; Riemann, 1985), especially when nutrients were also added (Fonte et al., 2011; Pace & Cole, 1996; Tzaras, Pick, Mazumder, & Lean, 1999). Other studies have investigated how lower trophic organisms, such as copepods, *Daphnia,* and nanoflagellates affect planktonic microbes (Birtel & Matthews, 2016; Jürgens, Arndt, & Rothhaupt, 1994; Wickham, 1998; Zöllner, Hoppe, Sommer, & Jürgens, 2009), revealing grazing‐mediated structural changes such as shifts to resistant morphotypes and changes in community composition. The majority of the studies focusing on fish effects, however, were performed before methods for the rapid characterization of bacterial diversity were developed (but see Wasserman, Matcher, Vink, & Froneman, 2015). Because microbial richness and community composition can have a profound effect on function (Leflaive et al., 2008; Peter et al., 2011), previous studies that have only investigated fish effects on bacterial production and biomass may, thus, have overlooked a suite of significant ecosystem effects.

Here, our goal was to investigate how a predator's presence, genotype (i.e., genetic background of fish), and phenotypic plasticity might influence bacterial communities. Our current study is part of a larger experiment, investigating the genotype and plasticity effects of the predatory stickleback (*Gasterosteus aculeatus*) on aquatic ecosystems (Matthews et al., 2016). Specifically, we used a lake–stream pair of stickleback from Lake Constance that has recently diverged into genetically distinct populations with differences in morphology and life history (Lucek, Roy, Bezault, Sivasundar, & Seehausen, 2010; Lucek, Sivasundar, & Seehausen, 2012; Marques et al., 2016; Roy, Lucek, Walter, & Seehausen, 2015). Such phenotypic divergence between lake and stream stickleback is common (Berner, Adams, Grandchamp, & Hendry, 2008; Lucek, Sivasundar, & Seehausen, 2014; Lucek et al., 2012; Marques et al., 2016), and often leads to diet differences where stream fish feed on benthic prey consisting of macroinvertebrates, whereas lake fish consume pelagic prey consisting of zooplankton (Lucek et al., 2012). In our specific population pair, some traits related to body shape were shown to be plastic (Lucek et al., 2014).

**Figure 1:**
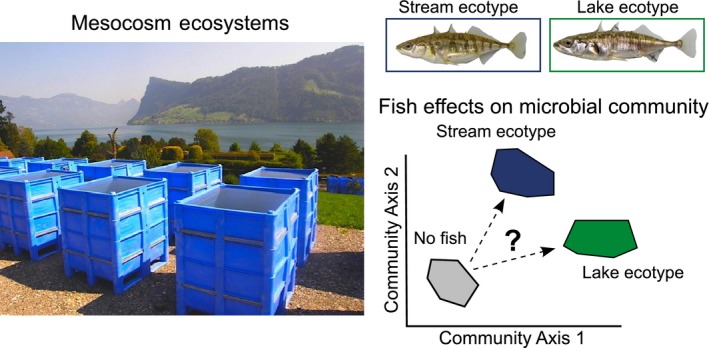
A schematic diagram illustrating the main experimental question from our experiment. We use experimental mesocosms to see whether the presence of stream or lake stickleback (*Gasterosteus aculeatus*) and their rearing diet affects the planktonic bacterial communities

Previously, Matthews et al. (2016) found that fish density (presence/absence), rearing environment, and genotype had a broad range of effects on ecosystem characteristics, ranging from effects on prey communities to ecosystem functions. In the current study, we focus on how bacterial communities responded to these same experimental manipulations. Specifically, we aimed to answer three main questions: (1) How does the effect of fish on the aquatic microbial community structure change through time? While the previous publication found, for one sampling date, that fish increased bacteria richness and abundance (Matthews et al., 2016), it did not investigate the changes in either richness or composition of bacteria through time. It also did not test putative drivers of bacterial communities (e.g., resources and grazers). (2) Are there interactive effects of genotype and rearing environment of fish on bacterial communities (Figure 1), as was previously found for both prey (e.g., zooplankton) and non‐prey (e.g., phytoplankton) communities in the same experiment (Matthews et al., 2016; Figure 1)? (3) Do the effects of fish presence and phenotype on the bacterial community persist or do they disappear after stickleback are removed and a homogenous fish treatment is added? We expected microbial communities to respond rapidly to changing environmental conditions, as shown in previous short‐term studies that manipulate zooplankton (Tranvik & Hansson, 1997). Overall, this work provides insights into how the presence and phenotype of fish can influence the planktonic microbial community.

## 2 Methods

### 2.1 Experimental setup

The current study, which was part of a larger experiment (Matthews et al., 2016), was designed to track temporal changes in the bacterial community through time in response to manipulations of the phenotype of a top predator. The full experiment used a randomized complete block design (*N* = 40 tanks), where each (spatial) block (*N* = 8) contained a tank without stickleback, and four tanks with stickleback (*Gasterosteus aculeatus)* representing four treatments made up of all factorial combinations of population origin (genotype: lake or stream) and rearing environment (benthic or pelagic food). Mainly for logistical reasons, we used six blocks (*N* = 30 tanks) to track changes in bacterial communities through time. The setup of the experiment is described in detail elsewhere (Matthews et al., 2016); here, we provide a brief summary. In 2010, we collected adult stream and lake ecotypes from Lake Constance, Switzerland, and set up mating trials for each ecotype (Matthews et al., 2016). The offspring of these matings were reared in the laboratory for 1 year, and were fed either a diet of either frozen bloodworms (*Chironomidae* sp. larvae) for the benthic diet, representing stream food, or zooplankton (*Daphnia* sp. and *Diaptomus* copepods) for the pelagic diet, representing lake food. These laboratory‐reared fish were then used for the outdoor mesocosm experiment (Figure 1). In April 2011, the mesocosms were set up and inoculated with plankton and benthic invertebrates from multiple lakes and streams, and on April 22, laboratory‐reared adult fish were added to the experiment (seven individuals per tank, except for the fish free tanks). This first phase of the experiment ended on August 18, when we began removing adults. The mesocosms were then fish free from August 25 through to September 13, at which point we added 18 juvenile lake fish and 18 juvenile stream fish to every tank. These juveniles were bred in the laboratory from wild adult populations and were raised for 16 weeks on a constant diet composed of a mix of plankton and chironomids prior to their addition to the tanks. This experimental design allowed us to test novel hypotheses about how the presence and absence of fish, as well as fish phenotype, could affect temporal patterns of bacterial richness and composition. In addition, on one of the sampling dates (5 August 2011), we used concurrent ecosystem measurements from Matthews et al. (2016) to test for putative drivers of bacterial richness and community composition. This was not possible for other sampling dates because no concurrent ecosystem measurements were available.

### 2.2 Sample collection

Over the course of the experiment, water samples for DNA extraction of planktonic microbes were taken on six sampling dates: 8 July 2011, 5 August 2011, 25 August 2011, 30 September 2011, 14 October 2011, and 27 October 2011. The first two sampling dates were taken while the adult stickleback were in 24 of the 30 tanks, the August 25th sampling point was taken while all mesocosm tanks were fish‐free, and the last three sampling points were taken after juvenile fish had been added to all mesocosm tanks.

For all sampling points, water was collected from the middle of the mesocosms in sterile glass bottles and placed on ice until filtration (within 5 hr) in the laboratory, where they were filtered using a vacuum manifold through sterilized filter holders. Volumes between 12 and 148 ml were directly filtered through a 0.2‐μm Supor^®^ membrane filter (Pall Scientific, USA). The amount of DNA used in ARISA runs was later standardized prior to analysis so that volume filtered would not influence our results. Filters were then frozen with liquid nitrogen and then stored in −80°C freezer until analysis. For flow cytometer measurements of microbial cell counts, additional water samples were taken and fixed in a final concentration of 0.01% paraformaldehyde and 0.1% glutaraldehyde and stored at 4°C for 4–8 months.

### 2.3 Flow cytometry of microbial cells

Our protocol followed Van Nevel, Koetzsch, Weilenmann, Boon, and Hammes (2013), but differed in that a 200‐μl aliquot of the sample was stained with 2 μl of SYBR^®^ Green I Nucleic Acid Gel Stain, 10.000× concentrated in DMSO (Invitrogen, USA) and incubated in the dark at 35°C for 5 min. A BD Accuri C6 flow cytometer was used to enumerate the microbial cells, and the number of particles was counted within a 50‐μl subsample. The flow rate was set to 35 μl/min, and the run duration was approximately 1 min 28 s. A threshold value of 500 was used for the green fluorescence channel (FL1). The BD Accuri CFlow^®^ software was used to process the data. We also employed a gating strategy similar to those previously described (Prest, Hammes, Kötzsch, van Loosdrecht, & Vrouwenvelder, 2013, SLMB, 2012). In short, a gate on the density plots of green (FL1; 533 nm) and red fluorescence (FL3; >670 nm) was used to distinguish microbial cells from instrument noise and sample background. Additional gating was used in the FL1/count histogram of microbial cells to differentiate smaller, low (LNA), and larger, high nucleic acid (HNA) microbes as described elsewhere (Van Nevel et al., 2013). The differentiation between HNA and LNA bacteria helps to characterize aquatic bacterial communities because HNA bacteria have larger genomes and are the more active bacterial constituents that account for most of the productivity, whereas LNA bacteria have smaller genomes and are thought to be less active (Lebaron, Servais, Agogué, Courties, & Joux, 2001). More recent work suggests that the LNA and HNA distinction between the bacteria based on flow cytometry also mirrors phylogenetic differences within communities (Vila‐Costa, Gasol, Sharma, & Moran, 2012).

### 2.4 DNA Extractions and amplification

The filters with planktonic microbes were cut into small pieces using sterile forceps and scissors. We used the same DNA extraction protocol as Czekalski, Sigdel, Birtel, Matthews, and Bürgmann (2015), except that the glass beads were 2 mm in diameter and samples were subjected to bead beating for 35 s at 5 m/s on a FastPrep^®^‐24 instrument. DNA was quantified with Quant‐iT Picogreen (Invitrogen) following manufacturer's protocols. Bacteria‐specific primers 1406F fluorescently labeled with 6FAM on the 5′ end and 23 Sr were used to target the intergenic spacer region of eubacteria between 16S and 23S rRNA (Fisher & Triplett, 1999). All PCR reactions were volumes of 25 μl with 5 ng of template DNA. A 1‐μl aliquot of PCR product that was diluted to 2.5 ng/μl to standardize the amount of DNA for the ARISA run was mixed with 9 μl highly deionized (HiDi) formamide and 0.5‐μl Liz1200 size standard (Applied Biosystems, USA). The mixture of PCR product, HiDi formamide, and size standard was denatured on a PCR thermocycler for 3 min at 95°C, and then placed on ice. A 3130XL Capillary Genetic Analyzer (Applied Biosystems) equipped with a 50 cm capillary using POP‐7 polymer was used for the denaturing capillary electrophoresis of each fragment. ARISA fragments between 200 and 1250 bp were analyzed with the Southern size‐calling method and a background cutoff level of 50 fluorescence units. ARISA peaks were binned using the automatic and interactive binning R scripts (Ramette, 2009) employing a binning window size of 2 bp, and the relative fluorescence intensity of binned peaks data was exported for further analysis. Blank extractions and amplifications were run with each set, and any peaks that were present in the blank samples were removed from all samples for further analysis.

### 2.5 Data analysis

ARISA peaks were interpreted as operationally defined taxonomic units (OTUs). The number of OTUs per profile was used to compare the bacterial diversity observable using the ARISA method. To examine the bacterial community composition (BCC), principal components were calculated using the Jaccard Index for presence/absence of bacterial OTUs and a Bray‐Curtis distance following a Hellinger transformation on abundance data that was based on ARISA peak area (normalized to total peak area per sample). *P* levels were established for model attributes based on 99,999 random permutations using a PERMANOVA test (Adonis). Linear mixed models with experimental block as a random effect were run on number of OTUs and flow cytometry data (microbial count and size). All statistical analyses were performed in R (v3.0.1; R Development Core Team 2013), using the vegan (Oksanen et al., 2013) and nlme (Pinheiro, Bates, DebRoy, & Sarkar, 2015) packages.

We tested whether variation in bacteria richness and community composition on August 5th could be explained by four ecosystem metrics (from Matthews et al., 2016), namely algal biomass (Chl‐a), zooplankton biomass, dissolved organic carbon (DOC), and bacterial abundance (from flow cytometry). For our analysis of bacterial composition, we used axes from a PCoA analysis, calculated with Bray‐Curtis distances using vegdist and cmdscale within the vegan package in R (Oksanen et al., 2013).

## 3 Results

### 3.1 Effect of fish on bacterial communities

**Figure 2:**
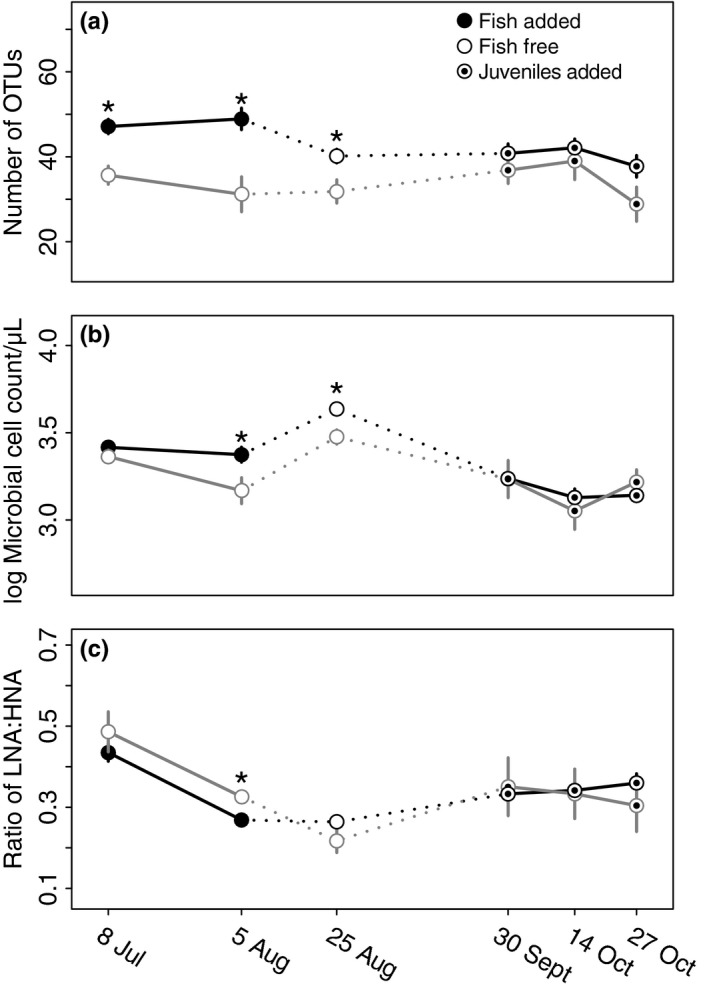
Mean microbial community characteristics in tanks with adult fish (filled circles), no fish (empty circles), and juvenile fish (circles with dots) including (a) the number of bacterial OTUs based on number of ARISA peaks per sample, (b) the log10 microbial cell count, and (c) the ratio of low nucleic acid (LNA) to high nucleic acid (HNA) bacteria based on flow cytometry. An asterisk indicates significant differences between the treatments for the respective time point (*p* < .05). For fish‐free treatments *n* = 6, and *n* = 24 for fish treatments. Error bars represent standard error. Lines connect means of tanks that were with or without fish at the start of the experiment

**Figure 3:**
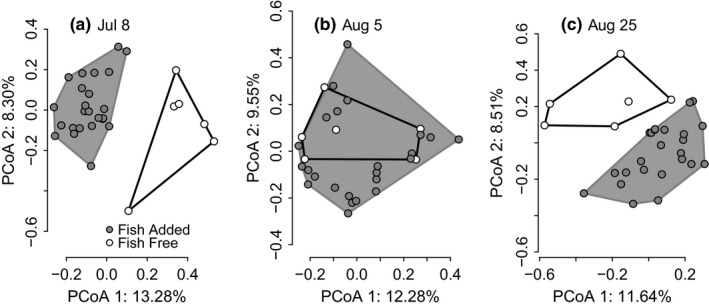
The effect of the presence of adult fish on bacterial community composition in lake mesocosms shown for the first two axes of a principal coordinates analyses based on abundance data derived from ARISA peak area from (a) July 8th, (b) August 5th, and (c) August 25th. Filled symbols indicate tanks with fish during the first two sampling points (July 8th and August 5th), and empty symbols represent tanks that received no fish. Both treatments had no fish on August 25th. For each sampling date, the shaded convex hulls outline the area for tanks that received fish and the non‐shaded convex hull indicates the area for tanks that did not receive fish

The presence of fish had significant impacts on number of OTUs, BCC, microbial cell count, and size structure (Table 1). The number of OTUs was significantly higher in tanks with stickleback during the two sampling dates, whereas fish were present in 24 of the 30 tanks (Figure 2a). The count of microbial cells was significantly higher and the ratio of LNA:HNA bacterial cells was significantly lower in tanks with fish during the second sampling date on August 5th than in tanks without fish (Table 1, Figure 2b,c). The presence of fish significantly affected BCC based on both presence/absence and abundance data for the first three sampling points (Table 1, Figures 3 and S1).

**Table 1:** Fish effects on mesocosm planktonic microbial communities. Flow cytometry (FCM) results showing effect of fish on microbial cell counts and the ratio of low nucleic acid (LNA) to high nucleic acid (HNA) microbes. ARISA results showing number of OTUs, and bacterial community composition based on presence and absence (BCCpa) and abundance (BCCa). F values are given with significant terms bolded. The degrees of freedom are (1,23) for ANOVA tests on FCM and the number of OTUs and (1,28) for Adonis analyses on BCCpa and BCCa

Date	Microbial cell counts and characteristics based on FCM		Bacterial community composition based on ARISA		
	Cell count	Ratio LNA:HNA cells	Number of OTUs	BCCpa	BCCa
Adult fish					
8‐July	0.68	2.03	**8.80** a	**2.74** a	**3.72** a
5‐August	**5.44** a	**7.53** a	**10.23** a	**1.48** a	**1.70** a
Fish removed					
25‐August	**6.24** a	2.33	**5.58** a	**2.00** a	**2.46** a
Juvenile fish added					
30‐September	0.00	0.10	0.67	0.85	0.8
14‐October	0.45	0.04	0.53	0.96	0.97
27‐October	1.00	2.03	2.61	1.05	1.02

a *p* < .05.

**Figure 4:**
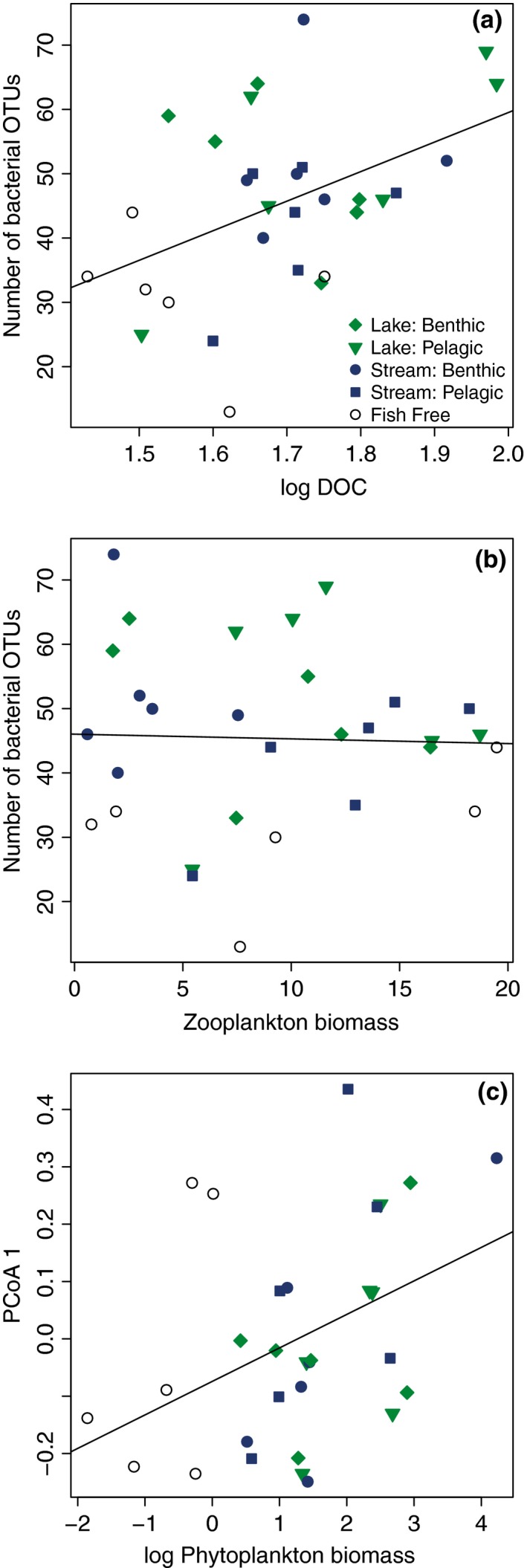
The relationship between selected ecosystem characteristics and bacterial community composition on August 5, including the number of bacterial OTUs compared to (a) dissolved organic carbon (DOC) and (b) zooplankton biomass, (c) and PCoA 1 based on community composition ARISA peak area and algal biomass. Black lines represent best linear fits for the correlations, which were significant (*p* < .05) in panels (a) and (c)

We used regression analyses to explore possible relationships between bacterial richness and composition and other ecosystem parameters (dissolved organic carbon [DOC], phytoplankton biomass (Chl‐a), zooplankton biomass, and bacteria abundance) on August 5th, and found that bacterial richness was positively correlated with dissolved organic carbon (*p* = .012), and moderately correlated with Chl‐a (*p* = .069), suggesting that the effect of fish on bacterial richness is most likely caused by changes in bacterial resources rather than via changes in zooplankton biomass (Figure 4). Furthermore, we explored the relationships between the first three principle components (34% of the variation) and Chl‐a, DOC, and zooplankton biomass, and we found that the first and third coordinates were positively correlated with Chl‐a (*p* = .024, *p* < .001, respectively) and the third was positively correlated with DOC (*p* = .002). We also found a positive relationship between bacterial abundance and phytoplankton biomass (*p* = .004).

### 3.2 Effect of fish genetic background and rearing history on bacterial community composition (BCC)

**Figure 5:**
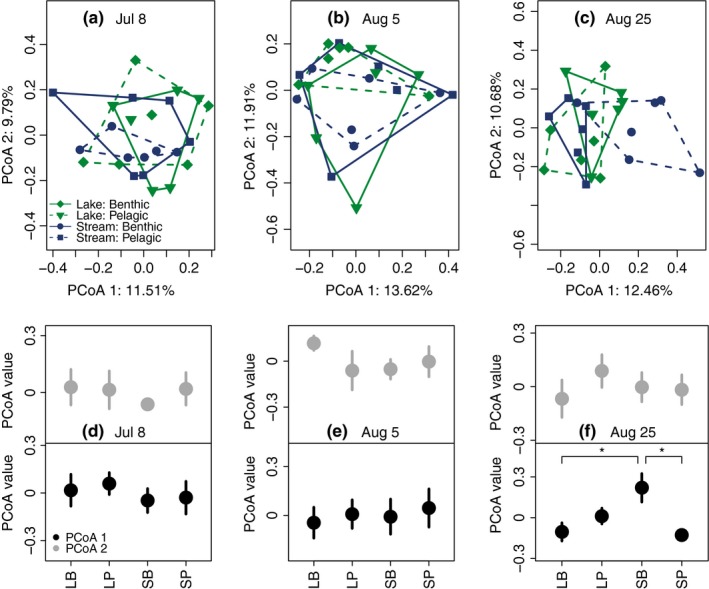
The effect of four different adult stickleback populations (treatments) on bacterial community composition using ARISA peak area on principal coordinates analysis from three sampling dates. For panels a–c, the data are colored by genetic background (Lake/Stream), and convex hulls indicating the area of each treatment are drawn with line types according to food (Benthic/Pelagic) upon which the fish were reared. In panels d–f, the first 2 PCoA coordinates from the four treatments are directly compared to each other to determine which treatments are driving G x E differences on August 5th and August 25th (see Table 2). In (d) July 8th, no differences are found among treatments, which was consistent with Adonis results. On (e) August 5th despite significant differences based on Adonis, Tukey's HDS test based on ANOVAs of showed no differences between PCoA axes 1 and 2. On (f) August 25th stream fish (S) on benthic food (B) appear to be driving differences in BCC (pairwise differences *p* < .05 are shown with asterisks)

On July 8th, we found no effect of genotype, rearing‐diet, or their interaction (i.e., phenotype effect) of adult stickleback on BCC, while we found a significant interaction effect of genotype × rearing diet on August 5th and August 25th (Table 2, Figure 5). On August 5th, the BCC of fish of different phenotypes was different based on Adonis (Table 2), since there are no post‐hoc tests designed specifically for use with Adonis, we ran Tukey's Honestly Significant Difference test on ANOVA results of the first two PCoA axes and did not identify pairwise differences among treatments (Figure 5b,e). On August 25th, the BCC found in tanks that had stream fish reared on benthic rearing diet minimally overlaped with the tanks of the other treatments and separated along the first principal component (Figure 5c,f). Fish phenotype did not affect the microbial cell count or number of bacterial OTUs for any of the sampling points. It did, however, influence the ratio of LNA:HNA bacteria (Table 3), and mesocosm tanks with fish reared on pelagic prey had a higher proportion of HNA bacteria on August 5th. When we compare the BCC of the tanks with adult stickleback (without the fish free tanks) to environmental parameters from the August 5th sampling date included in Matthews et al., 2016, we found that Chl‐a and the third principal component were positively correlated with each other (*p* = .004).

**Table 2:** Effect of genotype (G), food rearing diet (E), and their interaction (GxE) on planktonic bacterial communities in tanks that had fish added. ARISA results show the number of OTUs, and the bacterial community composition based on presence and absence (BCCpa) and abundance (BCCa). F values are given with significant terms bolded. The degrees of freedom are (1,15) for ANOVA test on the number of OTUs and (1,20) for Adonis analyses on BCCpa and BCCa

Date	Bacterial community composition based on ARISA								
	Number of OTUs			BCCpa			BCCa		
	G	E	G x E	G	E	G x E	G	E	G x E
Adult fish									
8‐July	0.99	0.23	0.49	0.94	0.86	1.02	0.79	0.81	1.14
5‐August	0.05	0.05	1.27	1.07	0.89	**1.16** b	**1.26** b	0.89	**1.33** b
Fish removed									
25‐August	1.85	2.73	3.12^a^	0.86	0.99	**1.59** b	0.94	1.02	**1.42** b
Juvenile fish added									
30‐September	0.00	0.02	0.19	0.89	0.96	0.80	0.94	0.89	0.75
14‐October	0.42	0.65	1.79	0.82	0.99	0.89	0.75	1.24^a^	0.87
27‐October	0.99	1.23	0.88	1.11	0.77	0.87	0.73	**1.42** b	0.78

a *p* = .1–.05.

b *p* < .05.

**Table 3:** Effect of genotype (G), food rearing diet (E), and their interaction (GxE) on planktonic microbial communities based on ANOVA tests. Flow cytometry (FCM) results showing effect of G, E and GxE on microbial cell counts and the ratio of low nucleic acid (LNA) to high nucleic acid (HNA) microbes. F values are given with significant terms bolded. The degrees of freedom are (1,15)

Date	Microbial cell counts and characteristics based on FCM					
	Cell count			Ratio LNA:HNA		
	G	E	G x E	G	E	G x E
Adult fish						
8‐July	0.31	0.28	0.43	0.07	0.09	0.69
5‐August	1.20	0.21	1.10	0.67	**5.82** b	2.63
Fish removed						
25‐August	0.12	4.05^a^	2.63	0.09	0.37	0.11
Juvenile fish added						
30‐September	1.48	1.00	2.01	0.46	0.19	1.00
14‐October	0.00	0.55	0.52	0.49	0.82	0.94
27‐October	0.69	2.07	0.85	3.41	0.09	1.05

a *p* = .1–.05.

b *p* < .05.

### 3.3 Persistence of effects

During the August 25th sampling, the microbial cell counts and number of OTUs remained higher in tanks that previously had fish compared to those that had no fish (Table 1). There were significant differences seen in BCC using both presence/absence and abundance data between fish and fish‐free tanks (Figure 3, Table 1). Fish phenotype effects on BCC were still apparent, as shown by a significant genotype × rearing diet interaction term (Table 2). The BCC of tanks that had stream fish reared on benthic food looked more divergent than the other treatment tanks (Figure 5c).

In contrast, for the sampling point on September 30th, which was about 2.5 weeks after the addition of the homogenous juvenile fish populations, we found no legacy effects of the prior presence or phenotype of fish (Tables 1 and 2). After the juvenile fish populations were added, no differences were observed among the previously fish‐free tanks and fish‐occupied tanks for the additional sampling periods (Table 1). BCC based on abundances was different between ecotypes at the last sampling point of the experiment (Table 2), but otherwise genotype and ecotype effects were absent once juvenile fish were added. Additionally, BCC shifted among sampling dates (Figure S2, *p* < .01 based on Adonis of BCC using abundance data).

## 4 Discussion

The presence of fish in aquatic systems has well‐documented effects on ecosystem structure and function, including alterations in zooplankton and phytoplankton communities and on nutrient and thermal dynamics (Carpenter & Kitchell, 1988; Chase, Biro, Ryberg, & Smith, 2009; Mazumder, Taylor, McQueen, & Lean, 1990; Vanni & Layne, 1997). Our results demonstrate that fish effects can also extend to the microbial community within the water column. While ARISA will almost certainly not capture the true diversity of the bacterial community and does not allow for taxonomic assignments of OTUs and/or phylogenetic information, it is a useful and frequently used parameter to compare bacterial community composition (BCC) (Bent, Pierson, & Forney, 2007), specifically in ecological time series and replicated environments (Bürgmann, Jenni, Vazquez, & Udert, 2011; Declerck, Winter, Shurin, Suttle, & Matthews, 2013).

Using this community‐fingerprinting approach, we found that fish presence had an impact on number of bacterial OTUs and BCC. Because fish have well‐documented effects on zooplankton size structure and abundance (Brooks & Dodson, 1965; Carpenter & Kitchell, 1988; Chase et al., 2009), fish‐mediated alterations of zooplankton communities could lead to subsequent changes in BCC due to cascading effects (Pace & Cole, 1996; Zöllner, Santer, Boersma, Hoppe, & Jürgens, 2003). However, on August 5th, variation in zooplankton biomass was neither affected by fish presence/absence nor was it correlated with changes in bacterial richness (Figure 4b). Instead, we found that fish increased DOC, which explained some of the variation (*R* ^2^ = .204) in the number of OTUs (Figure 4a). Based on the positive relationships between bacterial richness and DOC and bacterial composition and phytoplankton biomass, it seems likely that fish predators increased algal productivity and DOC, affecting the shifts in BCC. It has been suggested that microbial communities are regulated by nutrients rather than top‐down effects when nutrient levels are low (Pace & Cole, 1996). Indeed, previous studies have found that increased productivity can change microbial abundance, richness, and diversity although changes can be in different directions depending on the system (Horner‐Devine, Leibold, Smith, & Bohannan, 2003; Smith, 2007).

The effects of prior fish presence were apparent (Figures 2 and 3, Table 1) shortly after their removal. Because the generation times for planktonic bacteria at optimal conditions are several days (Wetzel, 2001), the slow turnover of planktonic microbes and the persistence of the ecosystem modifications (e.g., DOC pool, productivity) most likely contributed to measureable fish effects on August 25th. Following the addition of juveniles in the final stage of the experiment, the fish effects from earlier in the experiment disappeared. Unfortunately, without a sampling point toward the end of the fish‐free stage, we do not know whether the differences were already gone prior to the addition of the homogenous predator treatment. However, the additional data from these samplings confirm that experimental tanks that were previously subject to the fish/no fish treatments remained statistically indistinguishable under a homogenous treatment over the course of several weeks (Table 1). It should be noted that for this stage of the experiment we no longer had controls to differentiate the effect of the addition of juveniles from general, for example, seasonal effects. Such effects could have influenced the experiment since we found strong differences in BCC among sampling dates (Figure S2). Additionally, the reappearance of significant effects of phenotypic plasticity of fish on BCC during the last sampling (October 27th) could be due to the persistence of ecosystem effects from the phenotype treatments (Matthews et al., 2016) or due to an interaction with other ecosystem changes (i.e., changing seasons).

To our knowledge, no previous studies have tested for the effect of fish genotypes or of phenotypic plasticity on the temporal dynamics of microbial communities. In addition to a clear effect of the presence versus absence of predatory fish, the observed interaction effect of stickleback genotype × rearing diet on BCC (Figure 5, Table 2), thus, represents the first observation of a within‐species phenotype effect of fish on the BCC of planktonic bacteria. As the fish were not fed from external sources, once they were placed in the mesocosms, the difference in bacterial community structure arose because of differential ecosystem effects of the fish. This result suggests that the effects due to genotypic difference were modulated by the rearing conditions and give further support to a role of plasticity in influencing the ecosystem effects of fish (Lundsgaard‐Hansen et al., 2014; Matthews et al., 2016).

The interaction effect that we find between food rearing condition and genotype might help explain some of the indirect, non‐trophic effects that have been previously observed (Bassar et al., 2010; Harmon et al., 2009). The involvement of non‐consumptive, trait‐mediated effects can be on par with or greater than the effects of trophic interactions (Peacor & Werner, 2001; Preisser, Bolnick, & Benard, 2005). However, we were unable to detect the mechanisms that may account for this interactive effect with the environmental data from August 5th, and additional knowledge about the study system is needed to be able to pinpoint the mechanisms responsible for the interaction effect. We currently do not know how behavioral changes or dietary preferences from juvenile rearing conditions might change over time when faced with the diverse food sources in the mesocosms. Additionally, we did not quantify stoichiometric differences in diets. It is possible that phenotypic effects on bacterial communities could be affected by diet‐induced differences in fish stoichiometry (Vrede et al., 2011), thereby affecting nutrient dynamics. Alternatively, variation due to an animal's elemental phenotype (Jeyasingh, Cothran, & Tobler, 2014) may differ between locally adapted populations (Sullam et al., 2015; Tobler, Alba, Arias‐Rodríguez, & Jeyasingh, 2016) and could influence nutrient recycling. Therefore, more work is warranted on the stoichiometry of lake and stream stickleback.

Our analysis of BCC was done on a relatively coarse level using ARISA, which provides useful information on BCC, but does not allow phylogenetic identification of OTUs. Disentangling the mechanisms that control bacterial community composition would be assisted by a more detailed community analysis, for example, using next generation 16S rRNA gene amplicon sequencing or metagenomic, transcriptomic and proteomic analyses. These approaches could provide better information on diversity and additional information on the functional roles of affected microbial populations. For example, using 16S rRNA amplicon sequencing, Birtel and Matthews (In Press) found that manipulating the presence and absence of Daphnia in aquatic systems can dramatically alter the phylogenetic composition of the microbial community and shift the relative abundance of alpha and betaproteobacteria. Future research is needed to study how changes in both resource and grazer community dynamics affect the composition and functioning of bacterial communities.

## 5 Conclusion

Our study indicates, first, that the presence of fish markedly affects aquatic microbial community composition and structure by increasing the number of bacterial OTUs and the microbial cell counts. Second, when fish are present, genetic differences among closely related fish predators can have differential effects on aquatic microbial communities. It has been established that genotypes of organisms can affect the dynamics of their biological communities and ecosystem functioning (Whitham et al., 2003), and that such effects can arise through alterations of BCC and functioning (Madritch, Greene, & Lindroth, 2009; Schweitzer et al., 2011). While the existence of genotype‐specific effects of organisms on their environments has been documented, the plasticity of ecosystem effects has previously received minimal attention. Our results contribute to previous work using whitefish (Lundsgaard‐Hansen et al., 2014) and stickleback (Matthews et al., 2016) by showing that genotypic and plastic differences in fish predators can have effects that extend to the community structure of various levels within an aquatic ecosystem, including aquatic microbes. Therefore, it is important to assess how the origin of phenotypic variation (i.e., genetics or plasticity) of predators might affect trophic and non‐trophic interactions, and to study how predator‐mediated environmental modifications, including their effects on microbial community dynamics, may affect key biogeochemical processes such as carbon and nutrient cycling.

## Author Contributions

All authors contributed to the conception of the experiment. B.M., H.B, K.E.S., and T.A. carried out the experiment. B.M., H.B., and O.S. provided the infrastructure for experiments and laboratory work. K.E.S. did the analysis and wrote the manuscript with input and contributions from B.M. and H.B. All authors contributed to the editing and revising of the manuscript.

## Conflict of Interest

None declared.
