# Supplementary material for: The effect of top‐predator presence and phenotype on aquatic microbial communities
Source: Ecol Evol. 2017 Feb 8;7(5):1572–82. doi: 10.1002/ece3.2784 (PMC5330871; doi:10.1002/ece3.2784)

Supplementary Information for: The effect of top-predator presence and phenotype on aquatic microbial communities

Supplemental Figure 1: The 1st and 2rd (a to c) and the 1st and 3rd (d to f) dimensions of the PCoA represented in Figure 3 of the main part of the paper showing the effect of the presence of adult fish on bacterial community composition in lake mesocosms with the tanks numbered.

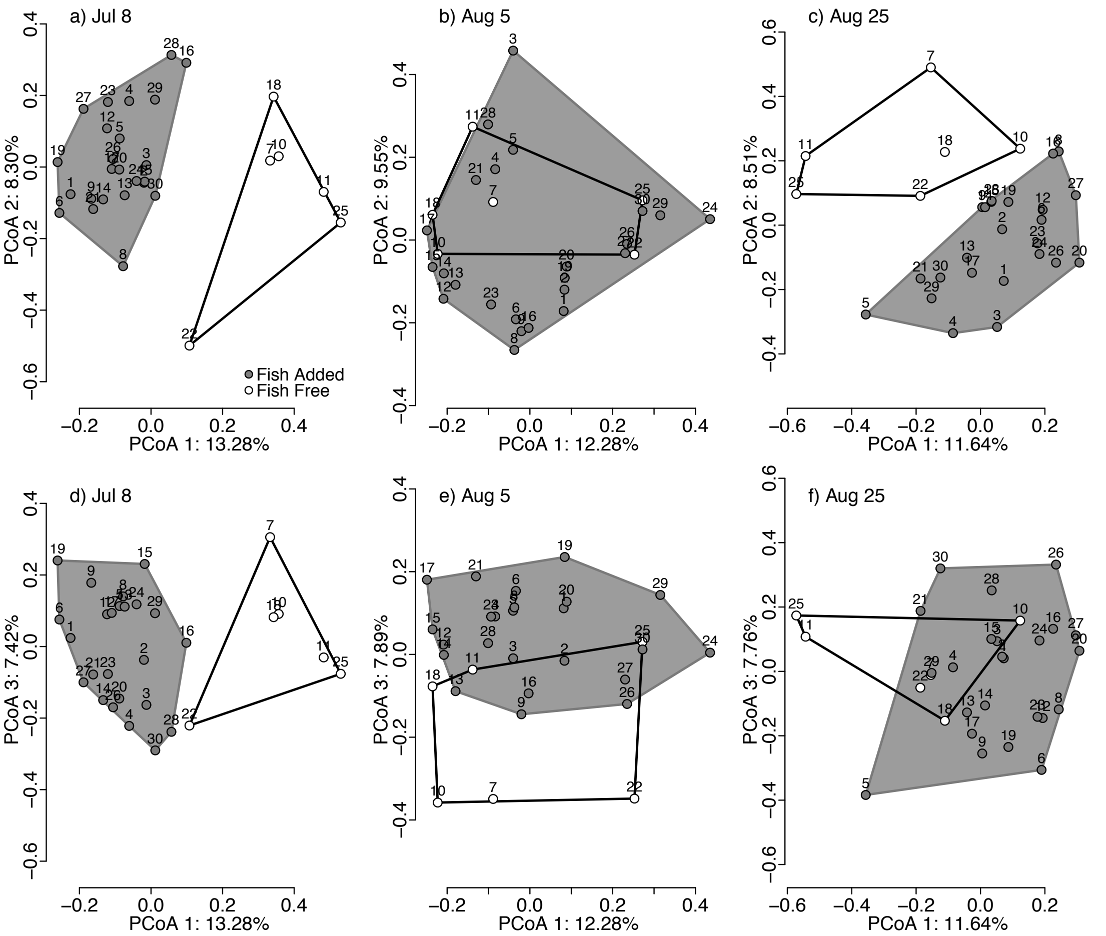

Supplemental Figure 2: Temporal development of bacterial community composition in mesocosm tanks, according to sampling date. Bacterial community composition is shown by non-metric multidimensional scaling on 3 dimensions with a) showing the 1st and 2nd dimension and b) showing the 1st and 3rd dimension. The first two sampling dates (July 8 and Aug 5) had treatments with and without adult fish, and shading for all time points corresponds to the presence of fish during these two dates. The adult fish had been removed from the tanks prior to August 25th. Prior to the last 2 sampling dates, juvenile fish were added all tanks.

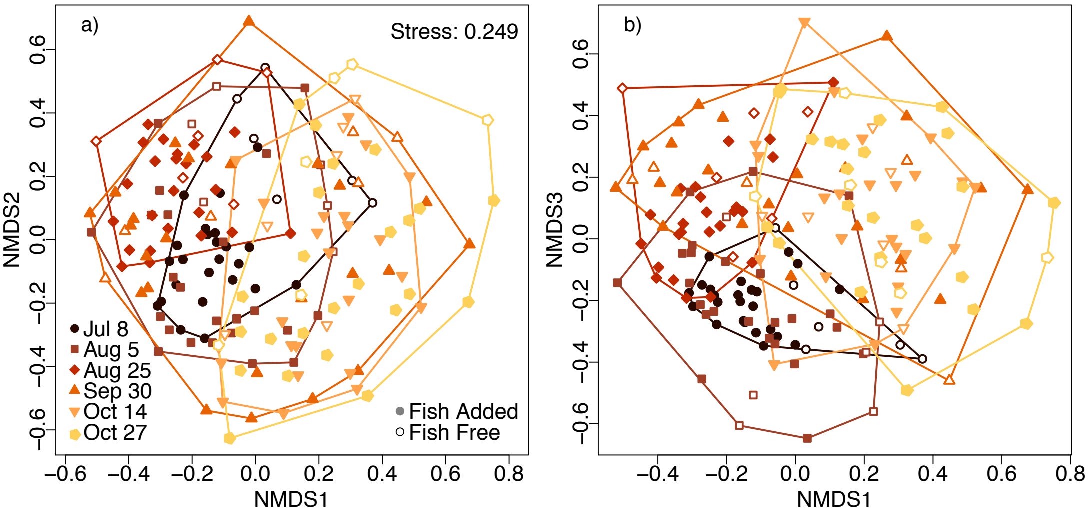

Supplement: Supplementary file 1 [file ECE3-7-1572-s001.pdf]
